# LncRNA SNHG1 promotes sepsis‐induced myocardial injury by inhibiting Bcl‐2 expression via DNMT1

**DOI:** 10.1111/jcmm.17358

**Published:** 2022-06-09

**Authors:** Rui Zhang, Zequn Niu, Jie Liu, Xiaoyan Dang, Hui Feng, Jiangli Sun, Longfei Pan, Zhuo Peng

**Affiliations:** ^1^ Emergency Department The Second Affiliated Hospital of Xi'an Jiaotong University Xi'an China

**Keywords:** B‐cell lymphoma‐2, DNA methyltransferase 1, inflammation, long noncoding RNA, methylation, myocardial injury, sepsis, small nucleolar RNA host gene 1

## Abstract

Myocardial injury is a frequently occurring complication of sepsis. This study aims to investigate the molecular mechanism of long noncoding RNA (lncRNA) small nucleolar RNA host gene 1 (SNHG1)‐mediated DNA methyltransferase 1/B‐cell lymphoma‐2 (DNMT1/Bcl‐2) axis in sepsis‐induced myocardial injury. Mice and HL‐1 cells were treated with lipopolysaccharide (LPS) to establish animal and cellular models simulating sepsis and inflammation. LncRNA SNHG1 was screened out as a differentially expressed lncRNA in sepsis samples through microarray profiling, and the upregulated expression of lncRNA SNHG1 was confirmed in myocardial tissues of LPS‐induced septic mice and HL‐1 cells. Further experiments suggested that silencing of lncRNA SNHG1 reduced the inflammation and apoptotic rate of LPS‐induced HL‐1 cells. LncRNA SNHG1 inhibited Bcl‐2 expression by recruiting DNMT1 to Bcl‐2 promoter region to cause methylation. Inhibition of Bcl‐2 promoter methylation reduced the inflammation and apoptotic rate of LPS‐induced HL‐1 cells. *In vivo* experiments substantiated that lncRNA SNHG1 silencing alleviated sepsis‐induced myocardial injury in mice. Taken together, lncRNA SNHG1 promotes LPS‐induced myocardial injury in septic mice by downregulating Bcl‐2 through DNMT1‐mediated Bcl‐2 methylation.

## 1 INTRODUCTION

Sepsis is a deadly event following major trauma leading to dysfunction of multiple organs, which is associated with infection‐caused extreme release of inflammatory factors into the blood.^1^, ^2^ Notably, myocardial dysfunction is a common condition in severe sepsis, involving key inflammatory mediators on both the heart and the peripheral vasculature.^3^ As a major complication to sepsis, septic cardiomyopathy is regarded as an important predictor for a poor clinical outcome in patients with sepsis.^4^ Weakened cardiac contractility and excessive cardiac inflammation, in addition to cardiomyocyte apoptosis, have also been implicated in the pathogenesis of sepsis‐induced cardiomyopathy.^5^

Intriguingly, the involvement of long noncoding RNAs (lncRNAs) in sepsis and sepsis‐induced cardiomyopathy has been highlighted by accumulating evidence.^6^, ^7^ Based on the bioinformatics prediction performed in the current study, the lncRNA small nucleolar RNA host gene 1 (SNHG1) was screened out as a differentially expressed lncRNA in sepsis. Notably, lncRNA SNHG1 has been involved in multiple pathologies including colorectal cancer cell growth,^8^ neurotoxicity in sevoflurane^9^ and oxygen‐glucose deprivation/reperfusion‐induced cell death.^10^ Emerging evidence has supported the protective role exerted by lncRNA SNHG1 in cardiomyocytes against hypertrophy,^11^ drug toxicity^12^ and apoptosis^13^; and it has further been suggested by Luo et al., that SNHG1 may alleviate sepsis‐induced cardiomyopathy through a microRNA (miRNA)‐dependent regulatory mechanism.^14^ On this basis, this study sought to further probe the role and mechanistic actions of lncRNA SNHG1 in sepsis‐induced cardiac injury.

As previously reported, lncRNA SNHG1 promoted gastric cancer cell proliferation by elevating the expression of DNA methyltransferase 1 (DNMT1).^15^ DNMT1 is regarded as a crucial gene being capable of regulating DNA methylation patterns in the process of genomic DNA replication.^16^ Intriguingly, increased total DNMT messenger RNAs (mRNAs) were observed in extracellular vesicles (EVs) from patients with septic shock compared with those in EVs from controls or patients with sepsis.^17^ Further, in a mouse model of emphysema, DNMT1 has been highlighted to induce hypermethylation of the Bcl‐2 promoter and downregulate Bcl‐2, which is an anti‐apoptotic factor to repress cell apoptosis.^18^, ^19^ Of note, cardiomyocyte apoptosis has been indicated to result in continuous increase of ventricular dysfunction in heart failure, including cardiomyopathy, myocardial infarction and ischaemia‐reperfusion injury.^20^

Therefore, we made a hypothesis in the present study that lncRNA SNHG1 may orchestrate the DNMT1/Bcl‐2 axis to affect the sepsis‐induced myocardial injury.

## 2 MATERIALS AND METHODS

### 2.1 Ethical approval

This study was performed under the approval of the laboratory animal care committee of Xi'an Jiaotong University. The animal experiments were conducted in accordance with the guidelines for the care and use of laboratory animals.

### 2.2 Bioinformatics analysis

Sepsis‐related microarrays GSE9667 (containing 6 myocardium samples of septic mice and 3 of normal controls) and GSE4479 (containing 5 myocardium samples of septic mice and 5 of normal controls) were retrieved from the GEO database. The GEO2R online analysis tool was then utilized for the identification of differentially expressed lncRNAs with *p* < 0.05 as the screening threshold.

### 2.3 Cell treatment

HL‐1 mouse cardiac muscle cell line was purchased from Procell and resuspended and incubated in 10% FBS‐contained DMEM medium under 37°C, 5% CO_2_. Upon reaching 80%–90% confluence, cells were subcultured. The cells of the third passage (P3) were treated with 1 μg/ml lipopolysaccharide (LPS) to induce inflammatory reaction.

For lentiviral transduction, HEK293T cells were cultured in 10% FBS‐contained RPMI‐1640 complete medium and passaged every other day. Untreated HL‐1 cells were used as control, and LPS‐treated HL‐1 cells without subsequent treatments were referred to as the LPS group. Some LPS‐treated HL‐1 cells were further transduced with lentivirus‐carried short hairpin RNA (shRNA) targeting lncRNA SNHG1 (sh‐SNHG1), lncRNA SNHG1 overexpression vector (oe‐SNHG1) or corresponding negative control (NC), referred to as sh‐SNHG1, sh‐NC (lentivirus vector pLKO.3G; Addgene), oe‐SNHG1 and oe‐NC (lentivirus vector pLenti6; Invitrogen) groups. Other LPS‐treated HL‐1 cells were further treated with dimethyl sulfoxide (DMSO) or 5‐aza‐dC (a methyltransferase inhibitor) alone or in combination with oe‐SNHG1, referred to as DMSO, 5‐aza‐dC, SNHG1 + DMSO and SNHG1 + 5‐aza‐dC groups. Lentivirus (1 × 10^8^ pfu/ml) was added into the cells. Each group was transduced with the same multiplicity of infection (MOI = 5).^21^ DMEM containing 10% FBS, 100 U/ml penicillin and streptomycin (Gibco) was used to screen stably transduced HL‐1 cells. Transduction sequences are shown in Table S1.

### 2.4 RNA quantification and RT‐qPCR

The Trizol reagent (Invitrogen) was utilized to extract total RNA from tissues or cells, and the RNA concentration was determined. mRNA was reversely transcribed into complementary DNA (cDNA) by the ImProm‐II™ RT system kit (Promega). The reversely transcribed cDNA was diluted to 50 ng/μl, followed by fluorescence qPCR using SYBR Premixed ExTaq II Kit (Takara). Primers were synthesized by Shanghai Genechem, as shown in Table S2. With GAPDH as the housekeeping gene, the relative mRNA expression was calculated with the $2^{‐\Delta \Delta C_{T}}$ method.

### 2.5 Western blot analysis

Total protein was extracted from tissues or cells by RIPA lysis buffer containing PMSF. BCA kits (#A53225; Thermo Fisher Scientific Inc.) were used to quantify protein concentration. The protein (50 μg for uploading) was dissolved in 2 × SDS sample buffer and boiled at 10 min at 100°C. After SDS‐PAGE, separated proteins were transferred to a PVDF membrane, which was then blocked in 5% skimmed milk powder at room temperature for 1 h and incubated with diluted primary antibodies against Bcl‐2 (ab182858, 1: 2000, rabbit; Abcam), Bcl‐2 associated protein X (Bax) (ab32503, 1: 1000, rabbit, Abcam), cleaved caspase 3 (ab214430, 1: 5000, rabbit, Abcam), caspase 3 (ab184787, 1: 2000, rabbit, Abcam) and GAPDH (ab9485, 1: 2500, mouse, Abcam) overnight at 4°C, followed by 3 washes using Tris Buffered Saline with Tween‐20. The membrane was subsequently incubated with secondary antibodies against IgG, either goat anti‐rabbit (Abcam, ab205718, 1: 20000) or goat anti‐mouse (Abcam, ab205719, 1: 20000), at room temperature for 1 h. Enhanced chemiluminescence substrate (Millipore, Billerica, MA) was used for image visualization. The grey value of protein bands was analysed by Image J software.

### 2.6 CCK‐8 assay

Cells were collected, detached, resuspended (1 × 10^5^ cells/ml) and seeded into a 96‐well plate with 100 μl/well. Cells were routinely cultured overnight and treated with CCK‐8 solution (Beyotime). The cell viability was detected by CCK‐8 method at 24, 48 and 72 h after seeding. Next, 10 μl of CCK‐8 detection solution was added to the cells in each test, followed by 4‐h incubation. The absorbance at 450 nm was detected using a microplate reader (Invitrogen; Thermo Fisher Scientific, Inc.).

### 2.7 Establishment of a mouse model of LPS‐induced sepsis

C57BL/6 male mice aged 6–10 weeks were purchased from Beijing Vital River Laboratory Animal Technology Co., Ltd. and raised in a specific‐pathogen‐free animal room at constant temperature (25–27°C) under constant humidity (45%–50%). Eight mice as controls (referred to as the Normal group) were intraperitoneally injected with of PBS (10 mg/kg). Then, 48 mice were intraperitoneally injected with LPS (10 mg/kg, Sigma‐Aldrich) for the model establishment and classified into three groups (*n* = 16), which were injected with LPS alone, or pre‐treated with sh‐NC or sh‐SNHG1 48 h before LPS induction, referred to as LPS, LPS + sh‐NC and LPS + sh‐SNHG1 groups.

Specifically, 48 h before LPS treatment, lentiviral vectors containing sh‐NC or sh‐SNHG1 (5 × 10^8^ pfu/100 μl; pSIH1‐H1‐copGFP, System Biosciences) were injected into corresponding mice by intraperitoneal injection.

Five hours after LPS or PBS injection, the mice were analysed by high‐resolution imaging system VisualSonics Vevo2100 (Visualsonics, Ontario, Canada). Left ventricular ejection fraction (EF) and fractional shortening (FS) were measured from the parasternal shortened M‐mode images. Echocardiographic data were recorded and analysed blindly. Among the 16 mice of each group, survival rate of 8 mice was recorded within 5 days, and the other 8 were euthanized 2 days after treatment by intraperitoneal injection of 50 mg/kg pentobarbital sodium to harvest the myocardium for subsequent experiments.

### 2.8 Echocardiography

The heart rate (HR) and ventricular function were evaluated by transthoracic echocardiography. The HR, left ventricular end‐diastolic diameters (LVEDD), left ventricular end‐systolic diameters (LVESD) and early to late mitral flow (E/A) ratio were measured for at least 3 consecutive times.

### 2.9 Flow cytometry

HL‐1 cells were seeded in a 6‐well plate at a density of 2 × 10^5^ cells/well. The supernatant was removed through centrifugation. The cells were then resuspended in PBS to adjust to a density of 1 × 10^5^ cells/ml. Cells were fixed in 75% ethanol at 4°C for 1 h. Following water bath at 37°C for 30 min in 100 μl of RNase A, 400 μl propidium iodide (PI) staining solution (Sigma‐Aldrich) was added to incubate cells at 4°C for 30 min under condition void of light. A flow cytometer (CytoFLEX S, Beckman Coulter) was applied to record the red fluorescence at excitation wavelength at 488 nm for cell cycle distribution.

After 48 h transfection, cells were detached with EDTA‐free trypsin and centrifuged to remove supernatant. With reference to the manual of an Annexin‐V‐fluorescein isothiocyanate (FITC) cell apoptosis detection kit (Sigma‐Aldrich), Annexin‐V‐FITC/PI staining solution was prepared by mixing Annexin‐V‐FITC, PI and HEPES at a ratio of 1: 2: 50 to incubate with the cells (1 × 10^6^ cells per 100 μl of solution) for 10 min at room temperature. After incubation with HEPES buffer, the fluorescence of FITC and PI was detected at 525 nm and 620 nm band‐pass filters at an excitation wavelength of 488 nm for cell apoptosis detection.

### 2.10 Enzyme linked immunosorbent assay

The levels of proinflammatory cytokines in HL‐1 cells were detected by corresponding ELISA kits for tumour necrosis factor‐α (TNF‐α) (ab181421, Abcam), interleukin‐1β (IL‐1β) (ab214025, Abcam) and IL‐6 (ab178013, Abcam).

### 2.11 Fluorescence in situ hybridization

Fluorescence in situ hybridization (FISH) was performed using FISH kits (Ribobio). cy3‐labelled lncRNA SNHG1 antisense probe 1 and lncRNA SNHG1 sense probe were provided by Ribobio, and cy3‐labelled lncRNA SNHG1 antisense probe 2 was provided by GenePharma. Cells were fixed in 4% formaldehyde for 15 min, permeabilized in PBS containing 0.5% TritonX‐100 at 4°C for 30 min and pre‐hybridized in pre‐hybridization solution at 37°C for 30 min. The probes were then added to the hybridization solution and incubated with the cells overnight at 37°C under condition void of light. On the next day, cells were stained with DAPI and photographed under a fluorescence microscope (BXF‐200; Bingyu Optical Instruments Co., Ltd.).

### 2.12 Nuclear‐cytoplasmic fractionation

The cytoplasmic and nuclear RNA purification kits (Norgen) were used for isolation and purification of cytoplasmic and nuclear RNAs. Polyadenylated RNA was captured with oligo (dT) polystyrene beads using GenEluteTM mRNA miniprep Kits (Sigma‐Aldrich).

### 2.13 Methylation‐specific PCR

Genomic DNA was extracted by a genomic DNA extraction kit (Beijing Tiangen Biochemical Technology). Concentration and purity of DNA were quantified by ultraviolet spectrophotometry, followed by storage in a refrigerator at −80°C. DNA was treated with sodium sulphite using EZ DNA methylation kits (Zymo Research Corp.), desulphurized and purified for subsequent PCR. Methylated and unmethylated primers were designed for the CpG island enrichment region of Bcl‐2 gene promoter (Table S3). The products were subjected to agarose gel electrophoresis. Image analysis was performed using a gel electrophoresis imaging analysis system. If the CpG islands in the promoter region of the gene were completely methylated, only the methylated primers could amplify the target bands. If completely unmethylated, only the unmethylated primers could amplify the target bands. If partially methylated, both pairs of primers could amplify the target bands. Partial methylation was classified into the methylation category. Grey value of target band was quantified using Image J software and the percentage of methylated and unmethylated DNA bands in total DNA bands was calculated.

### 2.14 RNA immunoprecipitation assay

RNA immunoprecipitation (RIP) assay was conducted with the use of Magna RIP RNA‐Binding Protein Immunoprecipitation kits (Millipore). Cells were lysed with 100 μl lysis buffer containing protease inhibitor and RNAase inhibitor on ice for 30 min, and centrifuged at 4°C and 12,000 ×*g* for 30 min. A small amount of the supernatant was taken as input positive control. Next, 1 μg of corresponding antibodies anti‐DNMT1 (1: 50, SC‐271729; Santa Cruz) and 10–50 μl protein A/G‐coated magnetic beads were added to the remaining supernatant for overnight incubation at 4°C. Following IP, the supernatant was removed through centrifugation at 4°C and 3000 ×*g* for 5 min. Protein A/G magnetic beads were precipitated with 1 ml lysate, followed by centrifugation at 4°C and 1000 ×*g* for 1 min. The RNA was isolated and purified from the precipitate by Trizol, and lncRNA SNHG1 expression was determined by RT‐qPCR.

### 2.15 RNA pull‐down assay

HL‐1 cells were harvested and lysed for 30 min with 100 μl of pre‐cooled lysis buffer containing protease inhibitor and RNAase inhibitor on ice for 30 min, followed by 30 min centrifugation (12,000 ×*g*, 4°C). The supernatant added with biotin‐labelled LncRNA SNHG1 probes (Ribobio) and incubated overnight with protein lysis buffer, followed by another 3‐h incubation at 4°C with M‐280 streptavidin magnetic beads (Sigma‐Aldrich). After that, the mixture was successively washed with cold lysis buffer, low salt buffer and high salt buffer to extract proteins, and DNMT1 expression was then determined using Western blot analysis. At the same time, an equal amount of cells were selected for lysis and the protein was extracted, and the expression of GAPDH was detected by Western blot as an internal reference protein. The grey value of protein expression in Western blot was determined with ImageJ software (NIH free software), the grey value of DNMT1 protein in each group was normalized with the grey value of GAPDH internal reference protein, and then, Input normalize to 1.0 to calculate the other two groups of DNMT1 protein grey value.

### 2.16 Chromatin immunoprecipitation

When the confluence of HL‐1 cells reached 70%–80%, 1% formaldehyde was added for fixation at room temperature for 10 min to crosslink DNA and protein. After crosslinking, the cells were ultrasonicated and then centrifuged at 13,000 rpm at 4°C to collect supernatant. The supernatant was, respectively, incubated with the NC antibody rabbit anti‐IgG (ab172730, 1: 100, Abcam) and the target protein specific antibody mouse anti‐DNMT1 (1: 50, SC‐271729; Santa Cruz) overnight at 4°C. The endogenous DNA‐protein complex was precipitated by Protein Agarose/Sepharose. The supernatant was removed through transient centrifugation, and non‐specific complex was rinsed. Following de‐crosslinking at 65°C, DNA fragments were purified and retrieved by phenol/chloroform. The binding of Bcl‐2 promoter with DNMT1 was detected. The specific primers for Bcl‐2 gene promoter were purchased from Ribobio.

### 2.17 TUNEL staining

TUNEL staining was used to detect apoptosis of HL‐1 cells according to the manual. The cells growing on coverslips were immersed with xylene, rehydrated in gradient ethanol and treated by proteinase K working solution. After preparation of TUNEL solution, cells were incubated with 50 μl of TUNEL reaction mixture (Beyotime) in a dark room at 37°C for 60 min. DAPI was used to incubate the slices at room temperature for 10 min, results of which were observed under a fluorescence microscope (BXF‐200; Bingyu). Green fluorescence was indicative of TUNEL‐positive cells, and blue fluorescence showed DAPI‐positive cells. The apoptotic rate was calculated by the ratio of TUNEL‐positive cells to DAPI‐positive cells.

### 2.18 Haematoxylin and eosin staining

The specimens fixed by formaldehyde were dehydrated, embedded into paraffin at 60°C, cut into 5‐μm slices and then dewaxed with xylene. After that, the slices were stained with HE reagents (Beijing Solarbio Biotechnology Co., Ltd.), dehydrated, permeabilized and observed using a fluorescent microscope (BXF‐200; Bingyu). Five different fields of view were selected for photographing, and then, ImageJ software was used to calculate the percentage of necrotic area in each field of view: necrotic area/total area × 100%.

### 2.19 Picrosirius red staining

The heart was isolated from euthanized mice, perfused with PBS, fixed in 4% paraformaldehyde for 8 h, dehydrated with 20% sucrose, embedded with paraffin and sliced into 5‐μm sections, followed by Picrosirius red staining to detect collagen deposition. The myocardial collagen deposition area fraction was calculated as the ratio of stained collagen deposition area to total myocardial area.

### 2.20 Statistical analysis

All the data in this study were analysed using the SPSS 21.0 statistical software (IBM). The quantitative data were expressed by mean ± standard deviation. Data between two groups were compared by independent sample *t* test, and those among multiple groups by one‐way analysis of variance (ANOVA) with Tukey's post‐hoc test. Variables were analysed at different time points using Bonferroni‐corrected repeated measures ANOVA. *p* < 0.05 indicated statistically significant difference.

## 3 RESULTS

### 3.1 LncRNA SNHG1 was highly expressed in myocardial tissues of LPS‐induced septic mice and cell model

**FIGURE 1:**
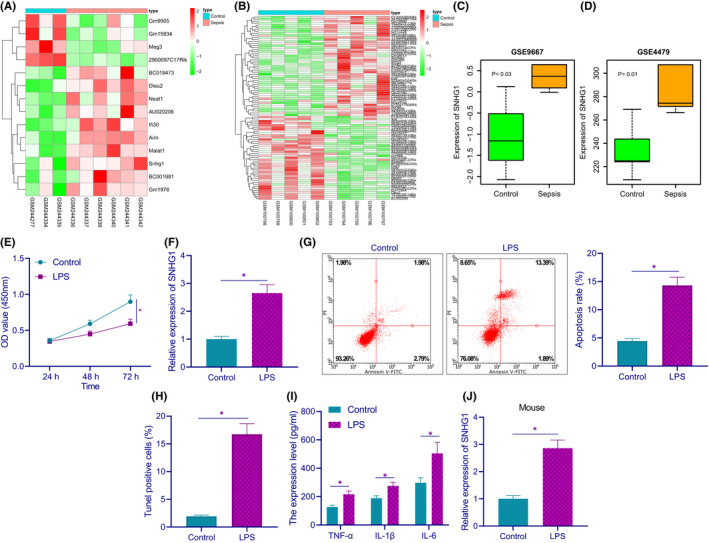
LncRNA SNHG1 is highly expressed in myocardial tissues of LPS‐induced septic mice and cell model. (A) The heatmap of differentially expressed lncRNAs in myocardial tissues between septic mice and control mice in GSE9667 expression dataset. (B) The heatmap of differentially expressed lncRNAs between septic mice and control mice in GSE4479 dataset. (C) The expression of lncRNA SNHG1 in septic mice in GSE9667 expression profile. (D) The expression of lncRNA SNHG1 in septic mice in GSE4479 expression profile. (E) HL‐1 cell viability in response to LPS induction. (F) The expression of lncRNA SNHG1 in HL‐1 cells in response to LPS induction. (G) Apoptotic rate in HL‐1 cells in response to LPS induction. (H) TUNEL positive rate in HL‐1 cells in response to LPS induction. (I) The production of TNF‐α, IL‐1β and IL‐6 in HL‐1 cells in response to LPS induction. (J) The expression of lncRNA SNHG1 in the myocardium of septic mice (*n* = 8). *p < 0.05. Cell experiment was repeated three times independently

Sepsis‐related datasets GSE9667 and GSE4479 were obtained from GEO database. Through microarray profiling, ten upregulated lncRNAs and four downregulated lncRNAs were identified in GSE9667 (Figure 1A). Meanwhile, 49 upregulated lncRNAs and 41 downregulated lncRNAs were screened from GSE4479 (Figure 1B). Among them, lncRNA SNHG1 was upregulated in both the two datasets (Figure 1C, D) and was thus selected for subsequent experiments.

In terms of the in vitro experiments, results of CCK‐8 assay showed that LPS‐treated cells had diminished cell viability (Figure 1E). RT‐qPCR results demonstrated that LPS‐treated cells presented with an increase in the expression of lncRNA SNHG1 (Figure 1F). Next, a notably increased apoptotic rate was found in LPS‐treated cells, as detected by flow cytometry and TUNEL staining (Figure 1G, H). ELISA results showed that LPS‐treated cells displayed increased production of inflammatory factors TNF‐α, IL‐1β and IL‐6 in LPS‐induced HL‐1 cells (Figure 1I).

The expression of lncRNA SNHG1 was subsequently determined by RT‐qPCR to be upregulated in the myocardium of LPS‐induced septic mice relative to that of control mice (Figure 1J). These results demonstrate that lncRNA SNHG1 is upregulated in myocardial tissue of LPS‐induced septic mice and cell model.

### 3.2 Silencing lncRNA SNHG1 reduced LPS‐induced inflammation and apoptosis in HL‐1 cells

**FIGURE 2:**
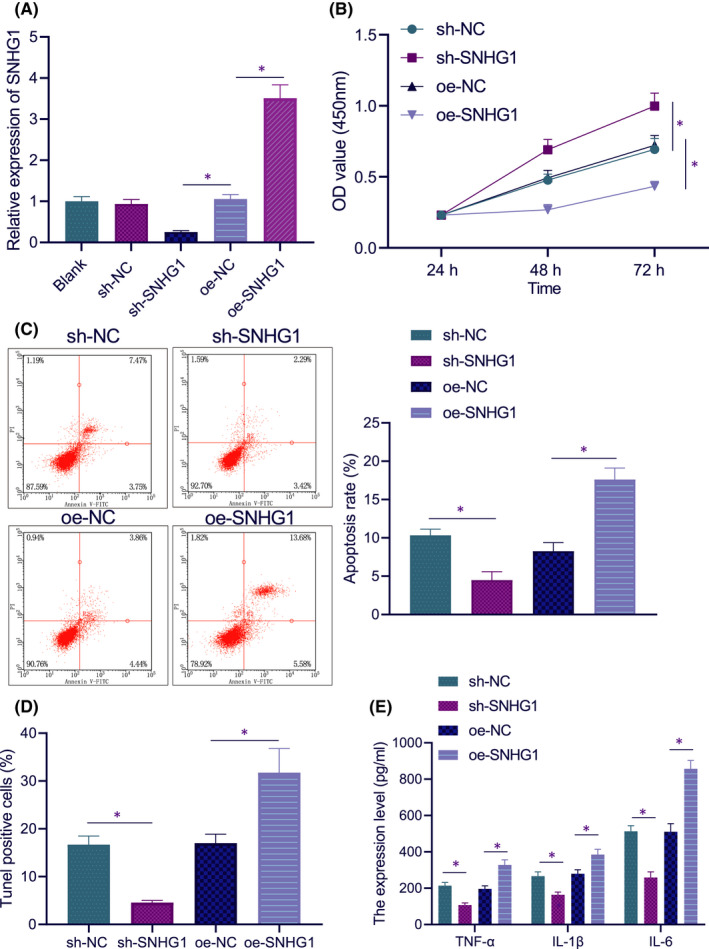
Silencing lncRNA SNHG1 reduces LPS‐induced inflammation and apoptosis in HL‐1 cells. (A) The expression of lncRNA SNHG1 in HL‐1 cells in response to sh‐SNHG1 or oe‐SNHG1. (B) HL‐1 cell viability in response to sh‐SNHG1 or oe‐SNHG1. (C) Apoptotic rate in HL‐1 cells in response to sh‐SNHG1 or oe‐SNHG1. (D) TUNEL positive rate in HL‐1 cells in response to sh‐SNHG1 or oe‐SNHG1. (E) The production of inflammatory factors in HL‐1 cells in response to sh‐SNHG1 or oe‐SNHG1. *p < 0.05. Cell experiment was repeated three times independently

To investigate the effect of lncRNA SNHG1 on LPS‐induced myocardial injury, we further infected LPS‐treated cells with lentiviral sh‐SNHG1/oe‐SNHG1, and the knockdown/overexpression efficiency was verified by RT‐qPCR (Figure 2A). The cell viability upon sh‐SNHG1 treatment was increased, while oe‐SNHG1 markedly diminished the cell viability, as determined by CCK‐8 assay (Figure 2B). In terms of the effect of lncRNA SNHG1 on apoptosis in myocardial injury, flow cytometry and TUNEL staining displayed that sh‐SNHG1 diminished apoptotic rate, while oe‐SNHG1 increased the apoptotic rate (Figure 2C, D). ELISA results showed that silencing lncRNA SNHG1 led to marked decreases in the production of inflammatory factors TNF‐α, IL‐1β and IL‐6, while lncRNA SNHG1 overexpression increased the production of these inflammatory factors (Figure 2E). These results suggest that silencing lncRNA SNHG1 reduces LPS‐induced inflammation and apoptosis in HL‐1 cells.

### 3.3 LncRNA SNHG1 inhibited Bcl‐2 expression through DNMT1

**FIGURE 3:**
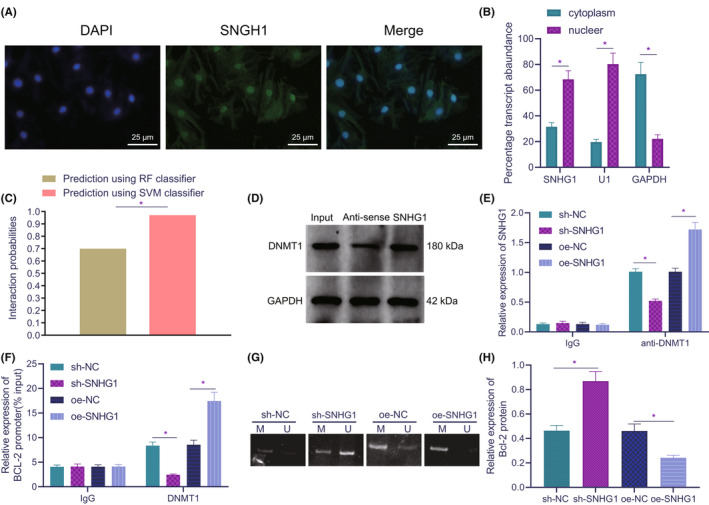
LncRNA SNHG1 inhibits Bcl‐2 expression through DNMT1. (A) Cell localization of lncRNA SNHG1. (B) The expression of lncRNA SNHG1 in the nucleus and cytoplasm, as normalized to U1 (nuclear positive control) and GAPDH (cytoplasmic positive control). (C) Probability of interaction between lncRNA SNHG1 and DNMT1 predicted by RPISeq online software (probabilities >0.5 are considered to be related). (D) The interaction between lncRNA SNHG1 and DNMT1. (E) Relative expression of lncRNA SNHG1 in presence of IgG or anti‐DNMT1 in response to sh‐SNHG1 and oe‐SNHG1. (F) Accumulation of DNMT1 on Bcl‐2 promoter in response to sh‐SNHG1 or oe‐SNHG1. (G) Methylation of Bcl‐2 promoter in response to sh‐SNHG1 or oe‐SNHG1. (H) The quantitative analysis for protein expression of Bcl‐2 in response to sh‐SNHG1 or oe‐SNHG1. *p < 0.05. Cell experiment was repeated three times independently

Subsequently, we investigated whether lncRNA SNHG1 can regulate the expression of DNMT1 in HL‐1 cells. FISH results unravelled that lncRNA SNHG1 was distributed in the nucleus and cytoplasm, but mainly in the nucleus (Figure 3A), which was further confirmed by nuclear‐cytoplasmic fractionation, similar to nuclear positive control U1 and different from cytoplasmic positive control GAPDH (Figure 3B). It was predicted by RPISeq online tool that lncRNA SNHG1 might bind to DNMT1 (Figure 3C). Hence, we speculated that lncRNA SNHG1 may interact with DNMT1.

To verify the speculation, we detected the interaction between lncRNA SNHG1 and DNMT1 by RNA pull‐down and RIP experiments in HL‐1 cells. The results of RNA pull‐down assay (Figure 3D) showed that lncRNA SNHG1 enriched DNMT1 protein, while GAPDH and magnetic beads alone could not enrich it. RIP experiment further confirmed the interaction between lncRNA SNHG1 and DNMT1. As shown in Figure 3E, overexpression of lncRNA SNHG1 resulted in increased binding between DNMT1 and lncRNA SNHG1, while silencing lncRNA SNHG1 led to opposite effects. These data confirmed the interaction between lncRNA SNHG1 and DNMT1.

We further explored the interplay among lncRNA SNHG1, Bcl‐2 methylation and DNMT1. ChIP assay results revealed that DNMT1 enrichment on Bcl‐2 promoter was increased in HL‐1 cells as compared with IgG, while silencing of lncRNA SNHG1 reduced the DNMT1 enrichment on Bcl‐2 promoter, which was reversed by overexpressing lncRNA SNHG1 (Figure 3F). In addition, the MSP experiments showed that the methylation of Bcl‐2 promoter in the presence of lncRNA SNHG1 knockdown was lower than that upon lncRNA SNHG1 overexpression (Figure 3G). Western blot analysis displayed that sh‐SNHG1 treatment led to increased Bcl‐2 protein expression and oe‐SNHG1 treatment led to the opposite results (Figure 3H, Figure S1A).

Overall, lncRNA SNHG1 downregulates Bcl‐2 expression through promotion on Bcl‐2 methylation by recruiting DNMT1 into Bcl‐2 promoter region.

### 3.4 Inhibition of Bcl‐2 promoter methylation reduced LPS‐induced inflammation and apoptosis in HL‐1 cells

**FIGURE 4:**
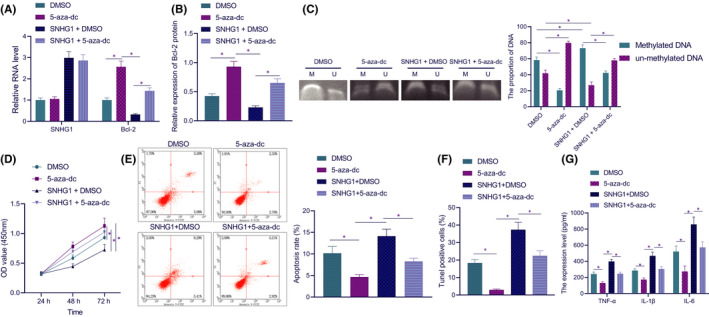
Inhibition of Bcl‐2 promoter methylation reduces LPS‐induced inflammation and apoptosis in HL‐1 cells. (A) The expression of lncRNA SNHG1 and Bcl‐2 in HL‐1 cells in response to lncRNA SNHG1 overexpression and 5‐aza‐dC alone or in combination. (B) The quantitative analysis for Bcl‐2 expression in response to lncRNA SNHG1 overexpression and 5‐aza‐dC alone or in combination. (C) Bcl‐2 promoter methylation in response to lncRNA SNHG1 overexpression and 5‐aza‐dC alone or in combination. (D) Detection of cell viability in response to lncRNA SNHG1 overexpression and 5‐aza‐dC alone or in combination. (E) Apoptotic rate of cells in response to lncRNA SNHG1 overexpression and 5‐aza‐dC alone or in combination. (F) TUNEL positive rate of cells in response to lncRNA SNHG1 overexpression and 5‐aza‐dC alone or in combination. (G) The production of TNF‐α, IL‐1β and IL‐6 in the supernatant of cells in response to lncRNA SNHG1 overexpression and 5‐aza‐dC alone or in combination. *p < 0.05. Cell experiment was repeated three times independently

In order to explore the role of lncRNA SNHG1 and Bcl‐2 in LPS‐induced inflammation and apoptosis in HL‐1 cells, we treated the cells with methyltransferase inhibitor 5‐aza‐dC. It was found that Bcl‐2 expression was elevated in 5‐aza‐dc‐treated cells, but reduced in SNHG1‐overexpressing cells As compared with SNHG1 overexpression alone, simultaneous treatment of SNHG1 and 5‐aza‐dC markedly increased the expression of Bcl‐2, according to results of RT‐qPCR and Western blot analysis (Figure 4A, B, Figure S1B).

Further, the MSP experiments suggested that the methylation of Bcl‐2 upon 5‐aza‐dc treatment was diminished, while the methylation of Bcl‐2 upon SNHG1 + DMSO displayed a marked increase. Relative to SNHG1 + DMSO, SNHG1 + 5‐aza‐dc contributed to diminished methylation of Bcl‐2 (Figure 4C).

The investigation focus was then shifted to the effect of Bcl‐2 promoter methylation on cardiomyocyte functions. CCK‐8 results displayed that the cell viability upon 5‐aza‐dC was increased, and that upon SNHG1 + DMSO was diminished. Compared with SNHG1 alone, its combination with 5‐aza‐dC resulted in increased cell viability (Figure 4D). Flow cytometric results showed that 5‐aza‐dC alone notably diminished the apoptotic rate, while additional SNHG1 overexpression increased apoptosis. Relative to SNHG1 overexpression alone, additional 5‐aza‐dC treatment diminished the apoptotic rate (Figure 4E). TUNEL staining results were consistent with flow cytometric results (Figure 4F). As shown by ELISA results, 5‐aza‐dC led to diminished production of TNF‐α, IL‐1β and IL‐6, which was reversed when lncRNA SNHG1 was simultaneously overexpressed. Relative to lncRNA SNHG1 overexpression alone, its combination with 5‐aza‐dC resulted in lower TNF‐α, IL‐1β and IL‐6 (Figure 4G).

These results suggest that inhibition of Bcl‐2 promoter methylation is capable of inhibiting HL‐1 cell inflammation and apoptosis.

### 3.5 Silencing of lncRNA SNHG1 in vivo alleviated sepsis‐induced myocardial injury in mice

**FIGURE 5:**
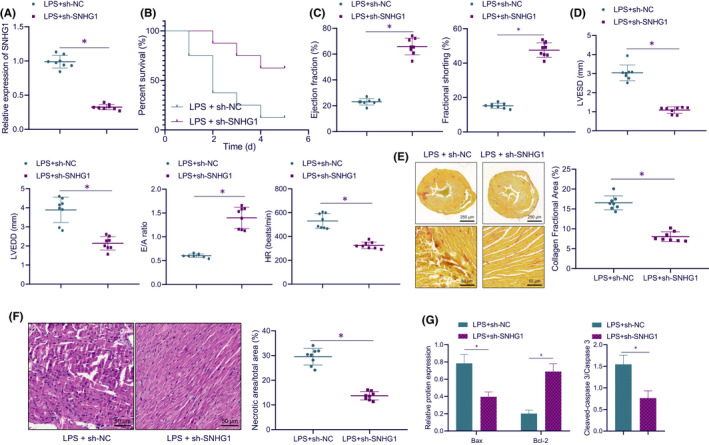
Silencing of lncRNA SNHG1 in vivo alleviates sepsis‐induced myocardial injury in mice. (A) Detection of lncRNA SNHG1 expression in myocardial tissues of mice. (B) The 5‐day mortality of mice in response to LPS alone or in combination with sh‐SNHG1. (C) The quantitative analysis of left ventricular EF and FS in mice in response to LPS alone or in combination with sh‐SNHG1. (D) The quantitative analysis of LVEDD, LVESD, E/A and HR in mice in response to LPS alone or in combination with sh‐SNHG1. (E) Collagen deposition area in left ventricular myocardial tissues in mice in response to LPS alone or in combination with sh‐SNHG1 (cross section of left ventricular myocardial tissues in the top panel; local amplification of left ventricular myocardial tissues in the bottom panel). (F) H&E staining was used to detect necrotic area in myocardial tissues of mice in response to LPS alone or in combination with sh‐SNHG1. (G) The quantitative analysis for expression of Bax, Bcl‐2, and cleaved caspase 3/caspase 3 in response to LPS alone or in combination with sh‐SNHG1. *p < 0.05. *n* = 8

To further explore the relationship between lncRNA SNHG1 and myocardial injury in septic mice, lncRNA SNHG1 was silenced in septic mice. LncRNA SNHG1 expression in the myocardium of septic mice was validated to be downregulated in response to sh‐SNHG1 treatment (Figure 5A). Moreover, sh‐SNHG1‐treated septic mice presented an increased 5‐day survival rate (Figure 5B). In addition, silencing lncRNA SNHG1 resulted in increased left ventricular EF and FS in LPS‐induced septic mice, based on ultrasonic cardiogram (Figure 5C). In the presence of sh‐SNHG1, HR, LVEDD and LVESD were decreased, while E/A ratio was elevated (Figure 5D). These results suggest that silencing lncRNA SNHG1 can improve myocardial function in LPS‐induced septic mice.

The results from Picrosirius red staining displayed that LPS induction led to larger collagen deposition area in left ventricular myocardium, while lncRNA SNHG1 knockdown reduced the myocardial collagen deposition area of LPS‐induced septic mice (Figure 5E). The results of HE staining revealed that in sh‐NC‐treated septic mice, myocardial tissues presented obvious degeneration and necrosis, swelling and breaking of partial nuclei, rupture of some myocardial fibres, blurring of myocardial cross stripes, oedema and increase of lipid in interstitial tissues. The aforementioned symptoms were alleviated, and the necrotic area in myocardial tissues was reduced in the presence of lncRNA SNHG1 silencing (Figure 5F). Moreover, lncRNA SNHG1 knockdown further led to diminished protein expression of Bax and cleaved caspase 3/caspase 3, while increasing that of Bcl‐2 in myocardial tissues of LPS‐induced septic mice (Figure 5G, Figure S1C).

These results suggest that silenced lncRNA SNHG1 in vivo can alleviate sepsis‐induced myocardial injury.

## 4 DISCUSSION

Myocardial injury is considered to be a frequently occurring symptom in sepsis.^22^ Unfortunately, sepsis‐induced myocardial injury presents a highly severe disorder for intensive care medicine because of its high morbidity and mortality.^23^ In this study, we investigated the role of lncRNA SNHG1 in sepsis‐induced myocardial injury and illustrated that lncRNA SNHG1 promoted sepsis‐induced myocardial injury by regulating the DNMT1/Bcl‐2 axis.

Our initial finding revealed that lncRNA SNHG1 was highly expressed in the LPS‐induced mouse and cell models, and that lncRNA SNHG1 knockdown reduced LPS‐induced inflammation and apoptosis in HL‐1 cells. Previous studies have suggested the role of lncRNA SNHG1 in pathogenesis of multiple disorders. For instance, intervention of lncRNA SNHG1 suppressed inflammation and apoptosis in HT22 cells, thereby decreasing sevoflurane‐stimulated neurotoxicity.^9^ In addition, downregulation of lncRNA SNHG1 was demonstrated to attenuate SH‐SY5Y cell death induced by oxygen‐glucose deprivation/reperfusion by functioning as a competing endogenous RNA for miR‐424.^10^ Of note, the regulatory relationship between lncRNA SNHG1 and DNMT1 has been reported by several studies. LncRNA SNHG1 elevated the expression of DNMT1, which promoted gastric cancer cell proliferation.^15^ In addition, Li *et al*. revealed that lncRNA SNHG1 contributed to enhanced development of liver cancer by decreasing p53 expression through binding to DNMT1.^24^ These reports support our finding that lncRNA SNHG1 recruits DNMT1 to affect sepsis‐induced myocardial injury.

Mechanistically, our study demonstrated that lncRNA SNHG1 recruited DNMT1 to promote Bcl‐2 methylation, which inhibited the expression of Bcl‐2. Intriguingly, accumulating evidence has unfolded the participation of DNMT1 in sepsis and myocardial injury. As previously reported, DNMT1 resulted in hypermethylation of miR‐145 promoter to downregulate miR‐145 expression, which promoted sepsis induced by LPS and shortened the overall survival of mice with sepsis.^25^ Wang et al. elaborated in their study that DNMT1 recruited by KCNQ1OT1 induced the methylation of RUNX3, which led to the downregulation of RUNX3 expression, thereby promoting cardiac microvascular endothelial cell injury as well as inflammatory reaction in acute myocardial infarction.^26^ Moreover, the use of DNMT inhibitors including procainamide and hydralazine contributed to alleviated hypotension, hypoglycaemia, as well as multiple organ dysfunction in rats with endotoxic shock induced by LPS.^27^ Importantly, the interaction between DNMT1 and Bcl‐2 has been increasingly reported. For example, DNMT1 contributed to hypermethylation of the Bcl‐2 promoter, which diminished the protein expression of Bcl‐2, thereby impairing lung function in a mouse model of emphysema.^18^ Moreover, E2‐mediated DNMT1 led to methylation of the Bcl‐2 promoter in Japanese eel ovarian follicle, thereby decreasing the expression of Bcl‐2.^28^ Of note, a prior study revealed that increased Bcl‐2 expression in mouse liver tissues following pretreatment with SDF‐1 aided in the therapeutic functions of endometrial regenerative cells on ameliorating sepsis symptoms.^29^ Upregulation of Bcl‐2 in rat lung tissues brought about reduced production of inflammatory cytokines and cell apoptosis in mice with sepsis‐induced lung injury.^30^ Overall, lncRNA SNHG1 regulated the DNMT1/Bcl‐2 axis to promote sepsis‐induced myocardial injury.

To conclude, our study demonstrated that lncRNA SNHG1 recruited DNMT1 to promote Bcl‐2 methylation, which diminished the expression of Bcl‐2, thereby promoting LPS‐induced myocardial injury in septic mice. These findings provide a theoretical basis for a novel direction for the treatment of sepsis‐induced myocardial injury. However, the clinical feasibility requires further verification.

## AUTHOR CONTRIBUTION

**Rui Zhang:** Conceptualization (equal); Investigation (equal); Methodology (equal); Visualization (lead); Writing – original draft (lead). **Zequn Niu:** Conceptualization (equal); Investigation (equal); Methodology (equal); Validation (lead); Writing – review & editing (equal). **Jie Liu:** Conceptualization (equal); Investigation (equal); Methodology (equal); Writing – review & editing (equal). **Xiaoyan Dang:** Data curation (equal); Formal analysis (equal); Software (lead); Writing – original draft (equal). **Hui Feng:** Data curation (lead); Formal analysis (equal); Writing – original draft (lead). **Jiangli Sun:** Data curation (equal); Formal analysis (equal); Writing – original draft (equal). **Longfei Pan:** Project administration (lead); Resources (equal); Writing – review & editing (equal). **Zhuo Peng:** Resources (equal); Supervision (lead); Writing – review & editing (equal).

## CONFLICT OF INTEREST

The authors declare that they have no conflict of interest.

## Supporting information

Fig S1

Table S1

Table S2

Table S3

Supplementary Material

## Data Availability

The data that supports the findings of this study are available in this article and its supporting information files.
