# Supplementary figures and images for: LncRNA SNHG1 promotes sepsis‐induced myocardial injury by inhibiting Bcl‐2 expression via DNMT1
Source: J Cell Mol Med. 2022 Jun 9;26(13):3648–58. doi: 10.1111/jcmm.17358 (PMC9258699; doi:10.1111/jcmm.17358)

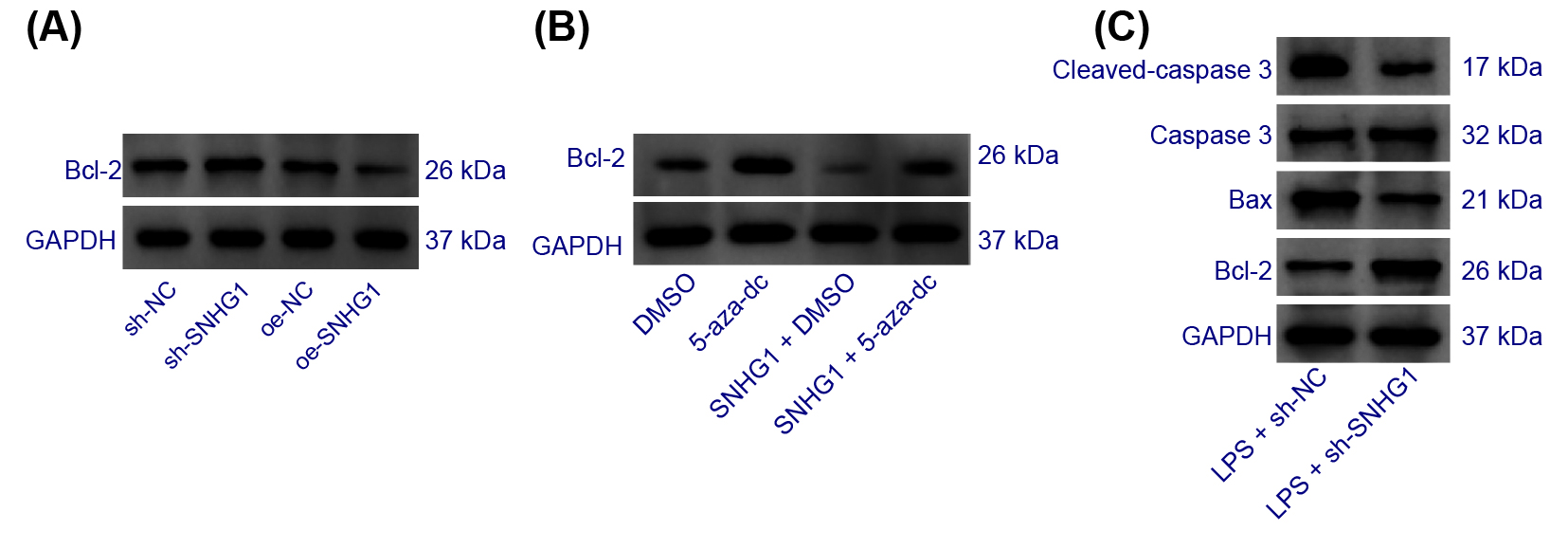

Supplement: Supplementary file 1 — Fig S1 [file JCMM-26-3648-s005.jpg]
