# Supplementary material for: LncRNA SNHG1 promotes sepsis‐induced myocardial injury by inhibiting Bcl‐2 expression via DNMT1
Source: J Cell Mol Med. 2022 Jun 9;26(13):3648–58. doi: 10.1111/jcmm.17358 (PMC9258699; doi:10.1111/jcmm.17358)
Supplement: Supplementary file 2 — Table S1 [file JCMM-26-3648-s002.docx]

**SUPPLEMENTARY TABLE 1** Transfection primer sequences

| shRNA | Sequence (5’-3’) |
| --- | --- |
| sh-NC | 5’-TTCTCCGAACGTGTCACGT-3’ |
| sh-SNHG1-#1 | 5’-CCGGACCATAAGAGATCACTTTAAACTCGAGTTTAAAGTGATCTCTTATGGTTTTTTG-3’ |
| sh-SNHG1-#2 | 5’-CCGGTGGAGCCAGGCCTGTTCAATTCTCGAGAATTGAACAGGCCTGGCTCCATTTTTG-3’ |
| sh-DNMT1-#1 | 5’-CCGGAGTGTGTGAGGGAGAAATTAACTCGAGTTAATTTCTCCCTCACACACTTTTTTG-3’ |
| sh-DNMT1-#2 | 5’-CCGGGAGTGTGTGAGGGAGAAATTACTCGAGTAATTTCTCCCTCACACACTCTTTTTG-3’ |

Note: sh- or shRNA, short hairpin RNA; NC, negative control; SNHG1, small nucleolar RNA host gene 1; DNMT1, DNA methyltransferase 1
