# Supplementary material for: LncRNA SNHG1 promotes sepsis‐induced myocardial injury by inhibiting Bcl‐2 expression via DNMT1
Source: J Cell Mol Med. 2022 Jun 9;26(13):3648–58. doi: 10.1111/jcmm.17358 (PMC9258699; doi:10.1111/jcmm.17358)
Supplement: Supplementary file 3 — Table S2 [file JCMM-26-3648-s004.docx]

**SUPPLEMENTARY TABLE 2** Primer sequences for RT-qPCR

| Gene | Sequence |
| --- | --- |
| SNHG1 | Forward: 5’-CCTTGTTCGGGGTTTGAGGT-3’ |
|  | Reverse: 5’-ACAGCACCCTGACTACAAGC-3’ |
| DNMT1 | Forward: 5’-CCGTGGCTACGAGGAGAAC-3’ |
|  | Reverse: 5’-TTGGGTTTCCGTTTAGTGGGG-3’ |
| Bcl-2 | Forward: 5’-GCTACCGTCGTGACTTCGC-3’ |
|  | Reverse: 5’-CCCCACCGAACTCAAAGAAGG-3’ |
| Bax | Forward: 5’-AGACAGGGGCCTTTTTGCTA-3’ |
|  | Reverse: 5’-AATTCGCCGGAGACACTCG-3’ |
| GAPDH | Forward: 5’-AATGGATTTGGACGCATTGGT-3’ |
|  | Reverse: 5’-TTTGCACTGGTACGTGTTGAT-3’ |

Note: SNHG1, small nucleolar RNA host gene 1; DNMT1, DNA methyltransferase 1; Bcl-2, B-cell lymphoma-2; Bax, Bcl-2-associated X protein; GAPDH, glyceraldehyde-3-phosphate dehydrogenase; RT-qPCR, reverse transcription-quantitative polymerase chain reaction
