# Supplementary material for: LncRNA SNHG1 promotes sepsis‐induced myocardial injury by inhibiting Bcl‐2 expression via DNMT1
Source: J Cell Mol Med. 2022 Jun 9;26(13):3648–58. doi: 10.1111/jcmm.17358 (PMC9258699; doi:10.1111/jcmm.17358)
Supplement: Supplementary file 4 — Table S3 [file JCMM-26-3648-s001.docx]

**SUPPLEMENTARY TABLE 3** Primer sequences for MSP

| Gene | Sequence |
| --- | --- |
| Bcl-2-M | Forward: 5’-CGAGGTGTTTAGGTTTTTTTTAGTC-3’ |
|  | Reverse: 5’-ACCCGCGCACTATATATAATACG-3’ |
| Bcl-2-U | Forward: 5’-TGAGGTGTTTAGGTTTTTTTTAGTT-3’ |
|  | Reverse: 5’-ACAACCCACACACTATATATAATACACT-3’ |

Note: Bcl-2, B-cell lymphoma-2; M, methylated; U, unmethylated; MSP, methylation-specific polymerase chain reaction.
