# Supplementary material for: LncRNA SNHG1 promotes sepsis‐induced myocardial injury by inhibiting Bcl‐2 expression via DNMT1
Source: J Cell Mol Med. 2022 Jun 9;26(13):3648–58. doi: 10.1111/jcmm.17358 (PMC9258699; doi:10.1111/jcmm.17358)
Supplement: Supplementary file 5 — Supplementary Material [file JCMM-26-3648-s003.docx]

**SUPPORTING INFORMATION**

**SUPPLEMENTARY FIGURE 1** Representative Western blots. A, The protein bands for expression of Bcl-2 in response to sh-SNHG1 or oe-SNHG1. B, The protein bands for Bcl-2 expression in response to lncRNA SNHG1 overexpression and 5-aza-dC alone or in combination. C, The protein bands for cleaved caspase 3, caspase 3, Bax and Bcl-2 in response to LPS alone or combined with sh-SNHG1.

**SUPPLEMENTARY TABLE 1** Transfection primer sequences. Note: shRNA, short hairpin RNA; NC, negative control; SNHG1, small nucleolar RNA host gene 1; DNMT1, DNA methyltransferase 1

**SUPPLEMENTARY TABLE 2** Primer sequences for RT-qPCR. Note: SNHG1, small nucleolar RNA host gene 1; DNMT1, DNA methyltransferase 1; Bcl-2, B-cell lymphoma-2; Bax, Bcl-2-associated X protein; GAPDH, glyceraldehyde-3-phosphate dehydrogenase; RT-qPCR, reverse transcription-quantitative polymerase chain reaction

**SUPPLEMENTARY TABLE 3** Primer sequences for MSP. Note: Bcl-2, B-cell lymphoma-2; M, methylated; U, unmethylated; MSP, methylation-specific polymerase chain reaction.
